# Prototyping and Experimental Analysis of Active Offloading Footwear for Patients With Diabetes Using an Array of Magnetorheological Fluid–Based Modules

**DOI:** 10.1177/19322968241260037

**Published:** 2024-06-17

**Authors:** Bhawnath Tiwari, Paolo Germano, Sarah L. Hemler, Christian Koechli, Zoltan Pataky, Yoan Civet, Yves Perriard

**Affiliations:** 1 Integrated Actuators Laboratory, Ecole Polytechnique Fédérale de Lausanne, Neuchâtel, Switzerland; 2 Unit of Therapeutic Patient Education, World Health Organization Collaborating Centre, University Hospitals of Geneva and University of Geneva, Geneva, Switzerland

**Keywords:** active offloading, diabetic foot ulcer, magnetorheological fluid, plantar pressure, smart footwear

## Abstract

**Background:**

Diabetic foot ulceration is a serious challenge worldwide which imposes an immense risk of lower extremity amputation and in many cases may lead to the death. The presented work focuses on the offloading requirements using an active approach and considers the use of magnetorheological fluid–based modules to redistribute high plantar pressures (PPs).

**Methods & Results:**

Experimentation validated a single module with a threshold peak pressure of 450 kPa, whereas an offloading test with a three-module array and complete footwear validated a maximum pressure reduction of 42.5% and 34.6%, respectively.

**Conclusion:**

To our knowledge, no such active and electrically controllable offloading footwear has been reported yet that has experimentally demonstrated PP reduction of more than 30% over the offloading site.

## Introduction

Globally, there are about 529 million people with diabetic mellitus and this number is expected to increase to 1.3 billion by 2050.^1^ In recent years, different complications^2^ either originating or linked with diabetes have been reported. One of the serious topics in these complications is Diabetic Foot Ulceration (DFU), which may bring potential threat to mobility as well as life of the concerned patients. About 50% of the population with a DFU are affected by lower extremity peripheral artery disease.^3^ Overall, there is a 50% five-year mortality rate following DFUs.^4^ Offloading^5^ is considered to be an effective method to take care of the ulcerated foot and in the prevention of DFUs. Removable offloading interventions are generally more preferred by the people with diabetes as it may provide better flexibility and mobility.^6,7^ However, non-removable offloading devices are considered to be more effective^8^ in DFU healing compared to removable options, but the user’s perceptions and experiences do not always align with this recommendation. This disparity indicates a strong need for further research to provide a convenient removable offloading alternative for people with diabetes.

As of recently, the majority of removable interventions are custom-made and are designed as fixed configuration systems^9-14^ or as reconfigurable (mechanical intervention) passive devices.^15^ As an alternative, this work aims to explore the offloading possibility of excess plantar pressure (PP) employing magnetorheological fluid (MRF) bringing electrical access for reconfiguration. We take advantage of the findings of our MRF-based previous work^16^ to develop an active offloading footwear, especially in terms of the valve capability to sustain ultra-high loads (>450 kPa) for an exposed area less than 100 mm^2^. The footwear is useful in maintaining a predefined safe PP for the prevention and/or care of DFUs. The offloading peak pressure limit may vary for both prevention and care (healing); thus, the electrical adjustability feature of the device could be useful in addressing both cases. Prototyping and experimental results are discussed, respectively, in sections “System Design, Working and Packaging Method” and “Experimental Works,” whereas section “Conclusions” presents the overall conclusion.

## System Design, Working and Packaging Method

For offloading in the present context, the key requirement is the ability to produce the negative stiffness profile in the deformation-reaction pressure scheme.^15^ To implement such an action, this work considers the use of arrays of the MRF-based offloading module (OM) so that the excess pressure can be shared (post-compression) and redistributed among modules having pressure lower than the peak pressure.

**Figure 1.**
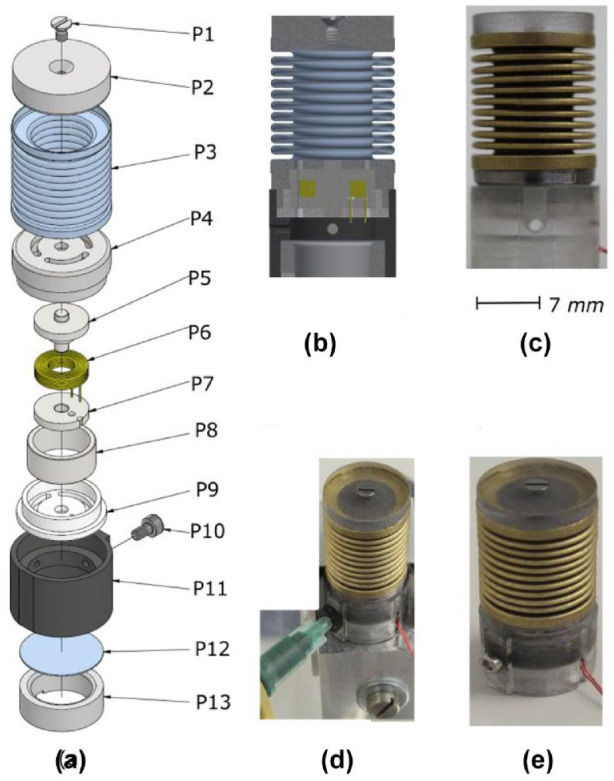
OM assembly and packaging (a) exploded view of assembly (b) assembled view (c) assembled prototype (d) MRF filling (e) ready to use OM.

The OM (Figure 1) consists of an MRF-based valve,^16^ a flexible silicone-based membrane (to allow compression once the valve is opened), and different supporting frames to accommodate the structure and its necessary flexibility. Different constituents are used according to the information provided in Table 1. The assembly is performed to provide the end product (OM) from these parts as shown in Figure 1.

**Table 1.** Different Parts Used in the Packaging of an OM.

Parts	Description
P1	M1, 6x3 stainless steel screw
P2	Laser Cut (PMMA)
P3	Metallic Bellow (Mera Bellows)
P4	3D Printed (PLA)
P5	Machined Shaft (Armco, *3dPCI*)
P6	Copper Coil (*Werap Wicklerei*)
P7	Machined Disk (Armco, *3dPCI*)
P8	Hollow Cylinder (Armco, *3dPCI*)
P9	3D Printed (PLA)
P10	M1, 6x3 stainless steel screw
P11	3D Printed (PLA)
P12	Silicone Disk (Elastosil, Wacker)
P13	3D Printed (PLA)

The current introduced (*ON* state) within the coil makes it act as an electromagnet, and therefore, the magnetization and so the threshold load limit can be defined by the inlet current. When the valve is opened (*OFF* state), the MRF passes through the cylindrical cavity (0.2 mm between P5 and P8) and pushes the flexible membrane (P12) leading to equivalent volume displacement of the fluid. This allows the metal bellow (P3) to be equivalently compressed and thus allows for offloading in our context.

**Figure 2.**
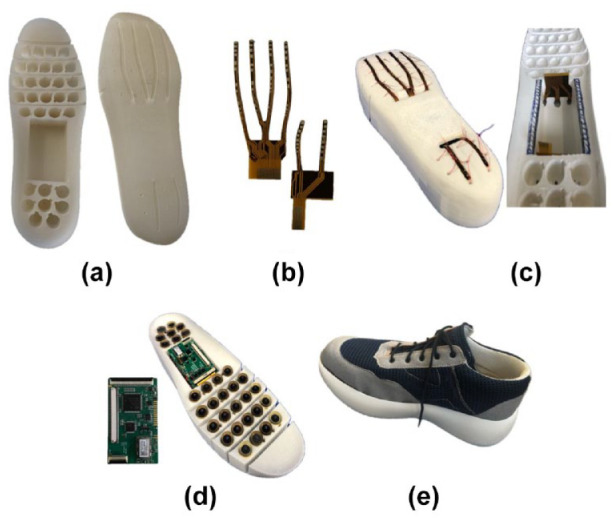
Packaging of complete shoe using the OMs and associated electronics: (a) flexible module fixation support, (b) flexible PCBs for connection to main (c) assembled flexible PCBs with fixation support, (d) main PCB, module packaging and connection, and (e) inserted offloading system in the footwear.

In the complete active offloading footwear (AOF), a flexible 3D printed module support (Figure 2a) compatible with the footwear (EU size 43) is used to hold an array of 31 OMs. Two flex printed circuit boards (PCBs) are positioned in the dedicated holes (between the forefoot and back foot region). There are, respectively, four and two finger-like ends of flex PCBs (Figure 2b and c) to allow the connection of OMs and other local components with the main PCB. The main PCB (Figure 2d) contains the global circuitry to perform *ON/OFF* action within the OMs and transfers the acquired data to the host PC. Circuit components and working scheme is detailed by Ntella et al.^17^

## Experimental Works

Experimental works are divided into three categories: first, the functioning of single OM is tested for its load withstanding/release capability; second, three OMs are used to validate the offloading working mechanism; and in the last part, an array of OMs embedded within the shoe is used to demonstrate the wearable device capability in enabling PP offloading.

### Single Offloading Module and Offloading With Three Offloading Modules

**Figure 3.**
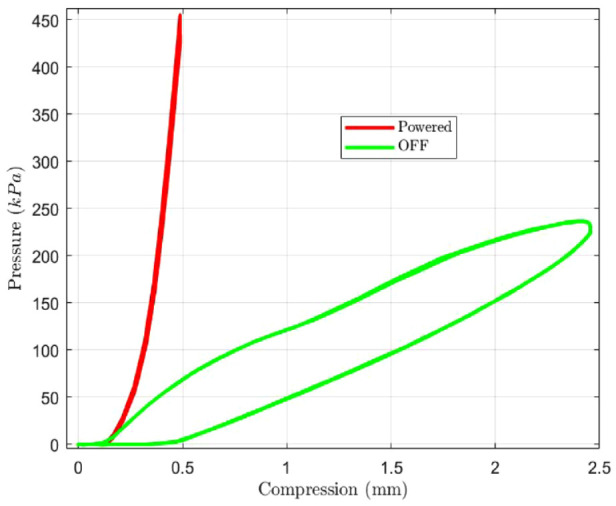
Load-withstanding capability of a single OM, powered (0.5 A) and unpowered behavior.

Ten loading cycles of 450 kPa were performed using a load applicator (ZTA—200N, IMADA, Toyohashi, Japan) on a single OM. The obtained behavior is shown in Figure 3, where the load-compression profile shows maximum 0.5 mm of compression (possible from air bubbles). Post-compression, the profile shows stable performance as evident from the repeated loading cycle.

**Figure 4.**
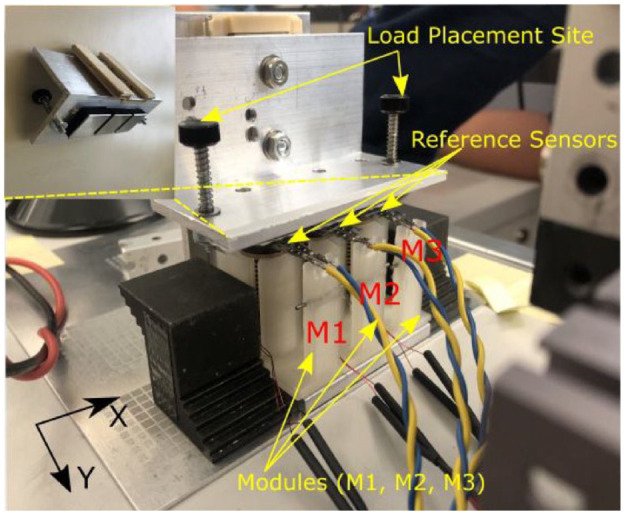
Experimental test bench for offloading validation using OM array.

For simplification of offloading analysis, three OMs (named M1, M2, and M3) were fixed equidistantly on a 3D printed holder (Figure 4), each mounted with reference sensors (Flexiforce A301, Tekscan, Norwood, MA, USA). The OM at the center (M2) was controlled to change the state (*ON/OFF*), whereas the other OMs M1 and M3 remained powered *ON* (current 0.5 A) during the experimentation.

**Figure 5.**
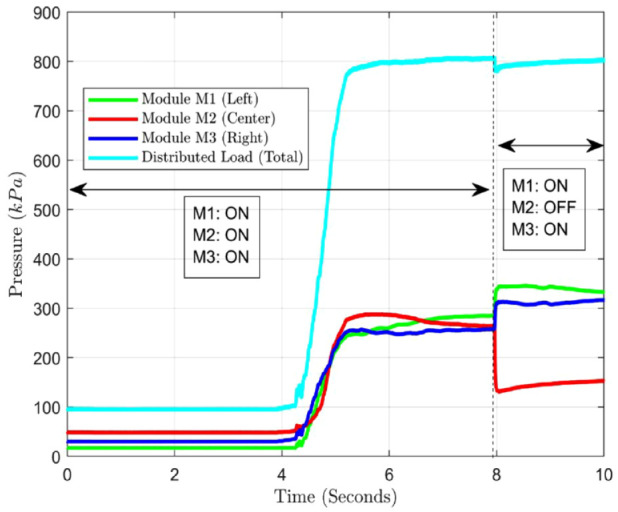
Offloading validation with three OM arrays.

A uniformly distributed load equivalent to 800 kPa was applied to the load placement site (Figure 4). Respectively, reference sensors of M1, M2, and M3 see an increase in pressure. The applied load (Figure 5) was then similarly distributed among the three OMs (∼265 kPa per OM) during the phase where all OMs were kept powered (*ON*). Post-turning *OFF* M2, M1, and M3 therefore underwent an increment in pressure (∼320 kPa), whereas M2 sees a decline to 152.4 kPa.

The total load (∼800 kPa) remained unchanged regarding pre-offloading and post-offloading which is in accordance with the experimentation (no loss of mass or energy exchange). The obtained offloading demonstrates a 42.5% decrease in pressure at the offloading site (M2 in the current case).

### Offloading From the Complete Footwear

**Figure 6.**
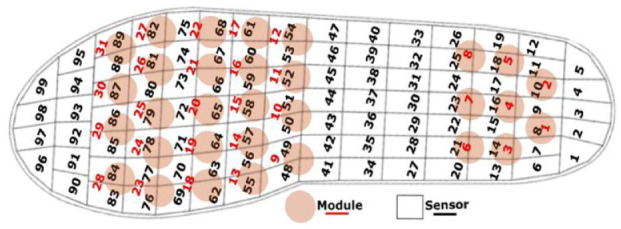
Relative positioning of *pedar-x* insole sensors and OMs.

A *pedar-x* sensing insole from *novelGmbH* (Munich, Germany) was inserted in the AOF (relative mapping shown in Figure 6) for the PP measurement and acquisition. The users of the AOF in the current experiments had no known foot abnormality, neither diagnosed with diabetes nor its complications in any form.

**Figure 7.**
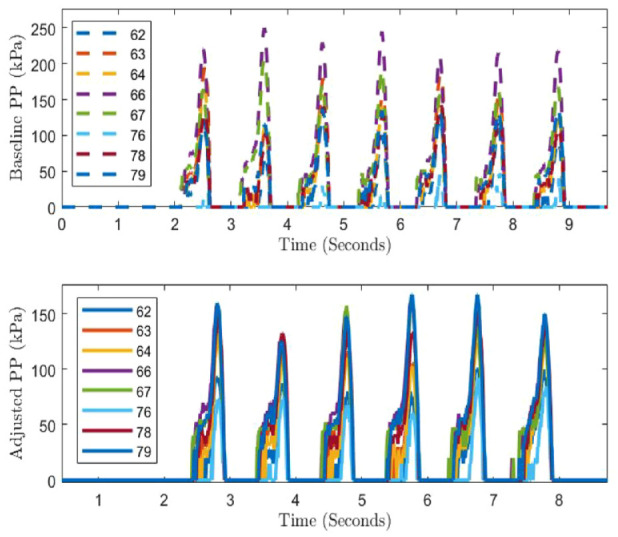
Baseline and adjusted profile corresponding to trial T6.

Six sets of experiments (T1, T2, T3, T4, T5, and T6) were conducted (Table 2); in each set, a baseline was considered as a reference, where all OMs were kept powered *ON* during the phase of user’s movement on a planar rigid surface (10 m). Out of six walking trials, five of them featured switching/control of single OM and one featured multiple OMs-based offloading (termed adjusted trials); for each trial, the baseline was performed separately. Baseline and adjusted PP profiles corresponding to T6 are shown in Figure 7 in which three OMs 18, 19, and 21 were switched *OFF* for offloading. Sensors 62, 63, 64, 66, 67, 78, and 79 of *pedar-x* were analyzed which cover the nearby region of the offloaded site.

**Table 2.** OM(s) Configuration for Different Trials.

Trial	OM(s) OFF	Maximum baseline peaks average (kPa)
T1	18	213.3
T2	23	273.3
T3	19	260.4
T4	19	308.5
T5	20	286.5
T6	18, 19, 21	226.4

**Figure 8.**
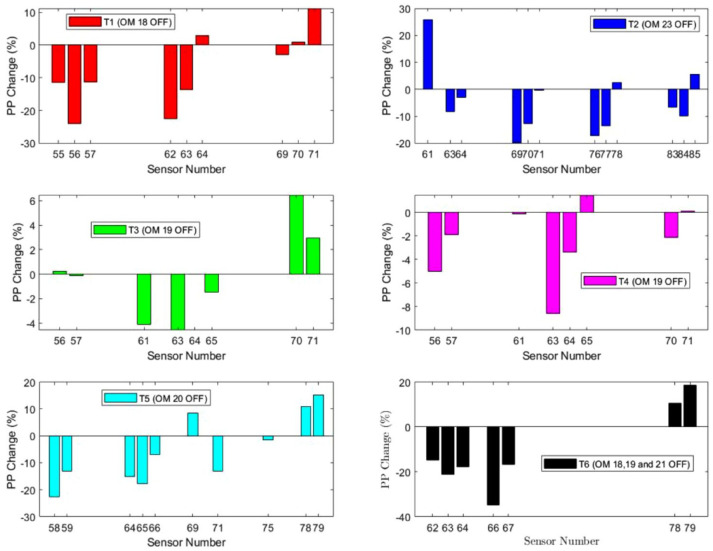
PP change in six different trials.

The average of the maximum PPs of each step from the baseline and from the adjusted (OMs *OFF*) trials was compared to compute the percentage change due to from the offloading. Different sensors of *pedar-x* which were in proximity (Figure 6) of the offloaded OM were studied for different trials (Figure 8).

Plantar pressure reduction (under and nearby offloaded OMs) and the shift to neighboring regions (local increment) were obtained. For different trials where a single OM was switched *OFF*, a maximum of 23.9% PP reduction was achieved (T1), whereas simultaneous deactivation of 3 OMs led to a maximum PP reduction of 34.6%. It is worth noting that the peak baseline PP in all the studies remained less than 350 kPa, and so a relatively low PP reduction was obtained for a single OM-based offloading action; however, a higher PP reduction may be achieved if multiple modules (such as of T6) are used for offloading. Plantar pressure increase of 3% to 25% was seen around the offloaded OM(s), which indicates the PP shift/distribution from the offloaded site(s).

## Conclusions

This work proposes a novel footwear dedicated to address the offloading needs using an array of active modules. A simplified offloading experiment featuring an array of three OMs has proven the capability of pressure reduction of 42.5% from the offloaded site, therefore validating the opted strategy of excess pressure distribution. The preliminary offloading outcomes using the developed AOF indicates its pressure offloading capability with more than 34% reduction in PP from the offloaded site when multiple OMs were manipulated simultaneously. More degrees of randomness in different trials, a better synergy among different OMs, and randomized clinical trials are additional steps which would be needed to complete before a possible recommendation to a person with diabetes can be made. Future work will aim to validate these said steps by employing an artificial intelligence–based decision-making for the offloading validation under controlled clinical conditions.
